# Supplementary material for: A living biobank of ovarian cancer ex vivo models reveals profound mitotic heterogeneity
Source: Nat Commun. 2020 Feb 13;11:822. doi: 10.1038/s41467-020-14551-2 (PMC7018727; doi:10.1038/s41467-020-14551-2)
Supplement: Supplementary file 1 — Supplementary Information [file 41467_2020_14551_MOESM1_ESM.pdf]

Supplementary Information

A living biobank of ovarian cancer ex vivo models reveals profound mitotic heterogeneity

Nelson et al.

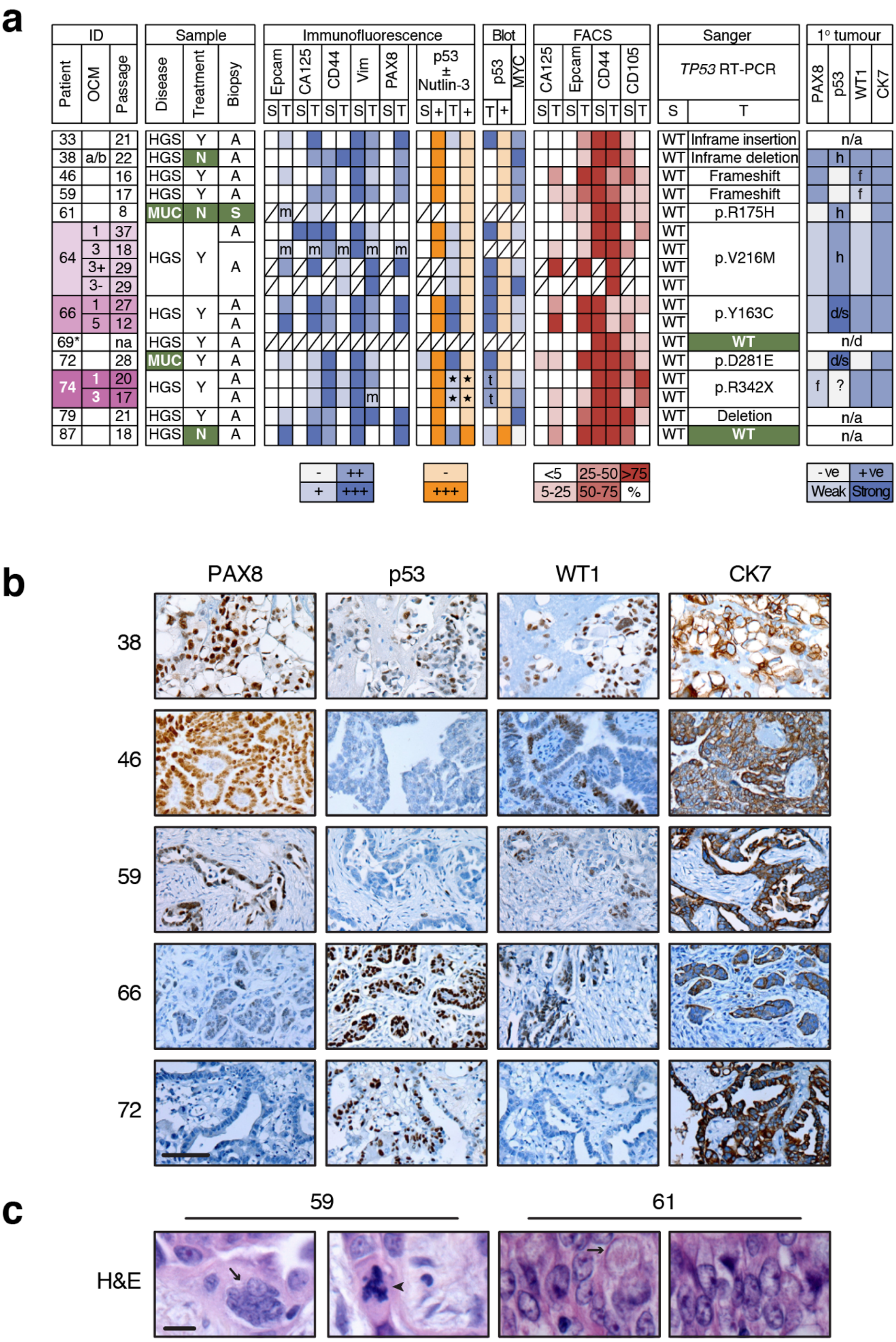

Supplementary Figure 1, related to Figures 1 and 2. Establishing and characterising *ex vivo* models. (a) Table summarising the cell biology data associated with the 12 patients and validation of the derived cultures. ID: specifies the patient and OCM sample numbers used throughout text. Note that culture established from the third biopsy from patient 64 was further separated into EpCAM negative and EpCAM positive cells, OCM.64-3^{Ep-} (64-3-) and 64-3^{Ep+} (64-3+), respectively. The passage number indicates the highest passage archived in

long term storage. Note that several have been cultured extensively beyond this, e.g. OCMs 38, 79 and 87 have been extended beyond passage 40. Note also that with the exception of OCMs 38a and 61, all the OCMs are recoverable from frozen stocks. Disease: HGS, high-grade serous ovarian cancer; MUC, mucinous ovarian cancer. Treatment: N, chemo-naïve. Biopsy: A, ascites; S, solid. Immunofluorescence: semi-quantitative analysis of the markers indicated, comparing stromal (S) and tumour (T) fractions. Asterisk indicates that OCM.69 is a stromal culture and not analysed by immunofluorescence. p53 ± Nutlin-3: blue indicates p53 detected in the absence of the Mdm2 inhibitor Nutlin-3; orange indicates induction by Nutlin-3, (-) no induction, (+++) strong induction; star indicates p53 aggregates. Blot: immunoblots of tumour fractions only (T) for p53, ± Nutlin-3, and MYC. (t), truncated. Colours as per immunofluorescence. FACS: flow cytometry quantitating the number of cells positive for the markers indicated. (m), mixed; (/), not done or not applicable. Sanger: *TP53* mutations identified by Sanger sequencing of RT-PCR products. WT, wild type. Analysis of the primary tumour summarizes the immunohistochemistry data generated by analysing the archival tumour blocks: (n/a), block not available; (f), focal staining; (h) wild type, heterogeneous staining; (d/s), diffuse strong staining; (n/d), not done; (?) undeterminable. (**b**) Representative 20x immunohistochemistry images of the primary tumours indicated stained to detect PAX8, p53, Cytokeratin 7 and WT1. Scale bar, 100 μm. (**c**) 63x H&E images of primary tumours showing a multi-nucleated giant cell (arrow) and a highly abberant mitosis in OCM.59, contrasted with mild atypia in OCM.61, where arrow highlights a mucin globule. Scale bar, 10 μm. Panels b and c are representative images from single experiment.

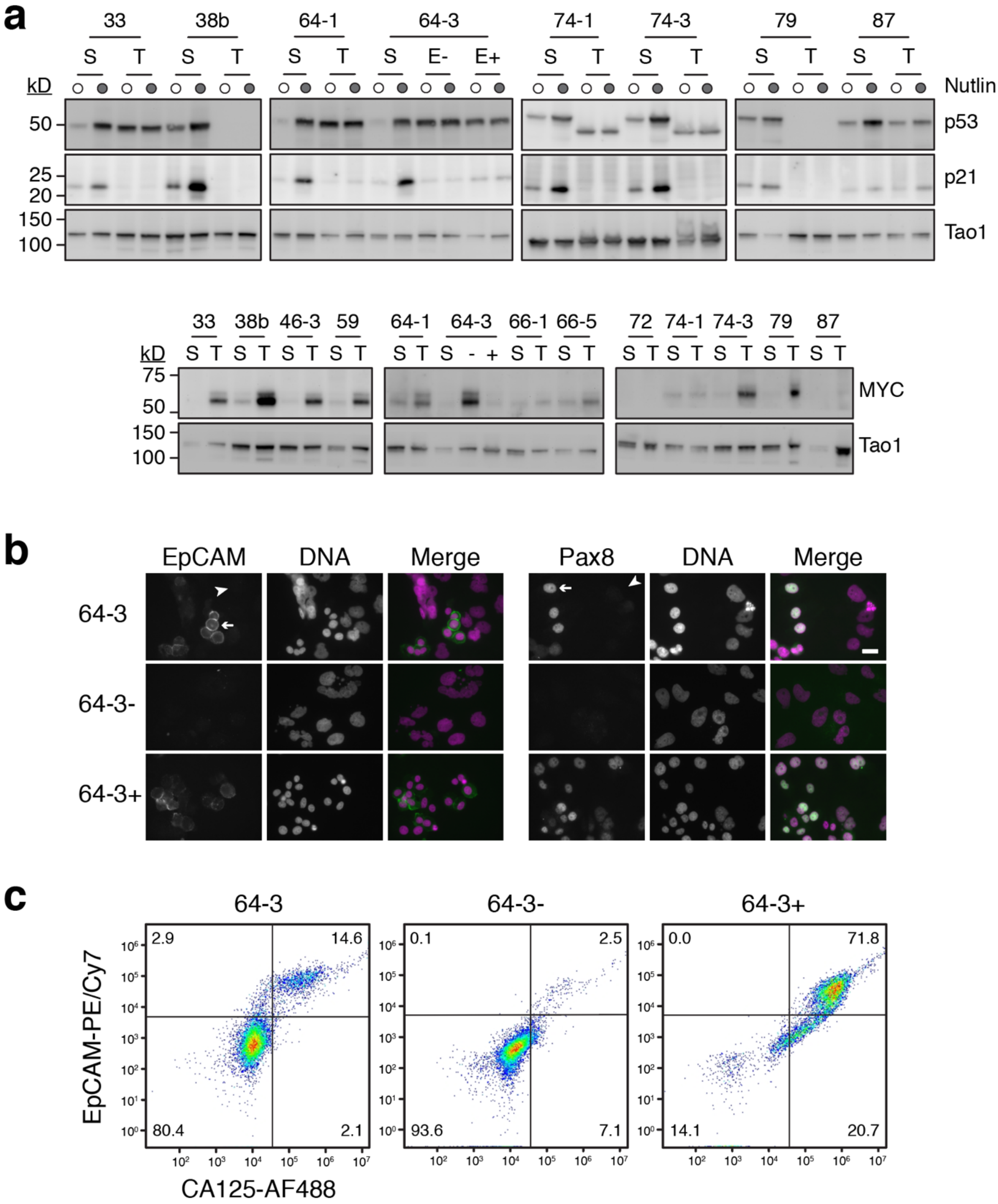

Supplementary Figure 2, related to Figure 2. Characterisation of *ex vivo* models. (**a**) Immunoblots showing expression of p53, p21 and MYC in stromal (S) and tumour (T) populations. p53 expression and its target p21 are analysed in the presence (closed circle) and absence (open circle) of the Mdm2 inhibitor Nutlin-3. Tao1 is used as loading control. (**b**) Immunofluorescence images of OCM.64-3, highlighting EpCAM/PAX8 negative cells (arrowheads) and EpCam/PAX8 positive cells (arrows). Scale bar, 20 μm. (**c**) Flow cytometry profiles of OCM.64-3 and the two sub-cultures generated by magnetic activated cell sorting (MACS) using anti-EpCAM antibodies coupled to microbeads, with 64-3- and 64-3+ denoting the EpCAM negative and positive populations respectively. Numbers represent the percentage of cells in the respective quadrant. Panels a and b are representative images from single experiment. Source data for panel a and c are provided as a Source Data file, including the gating/sorting strategy for panel c.

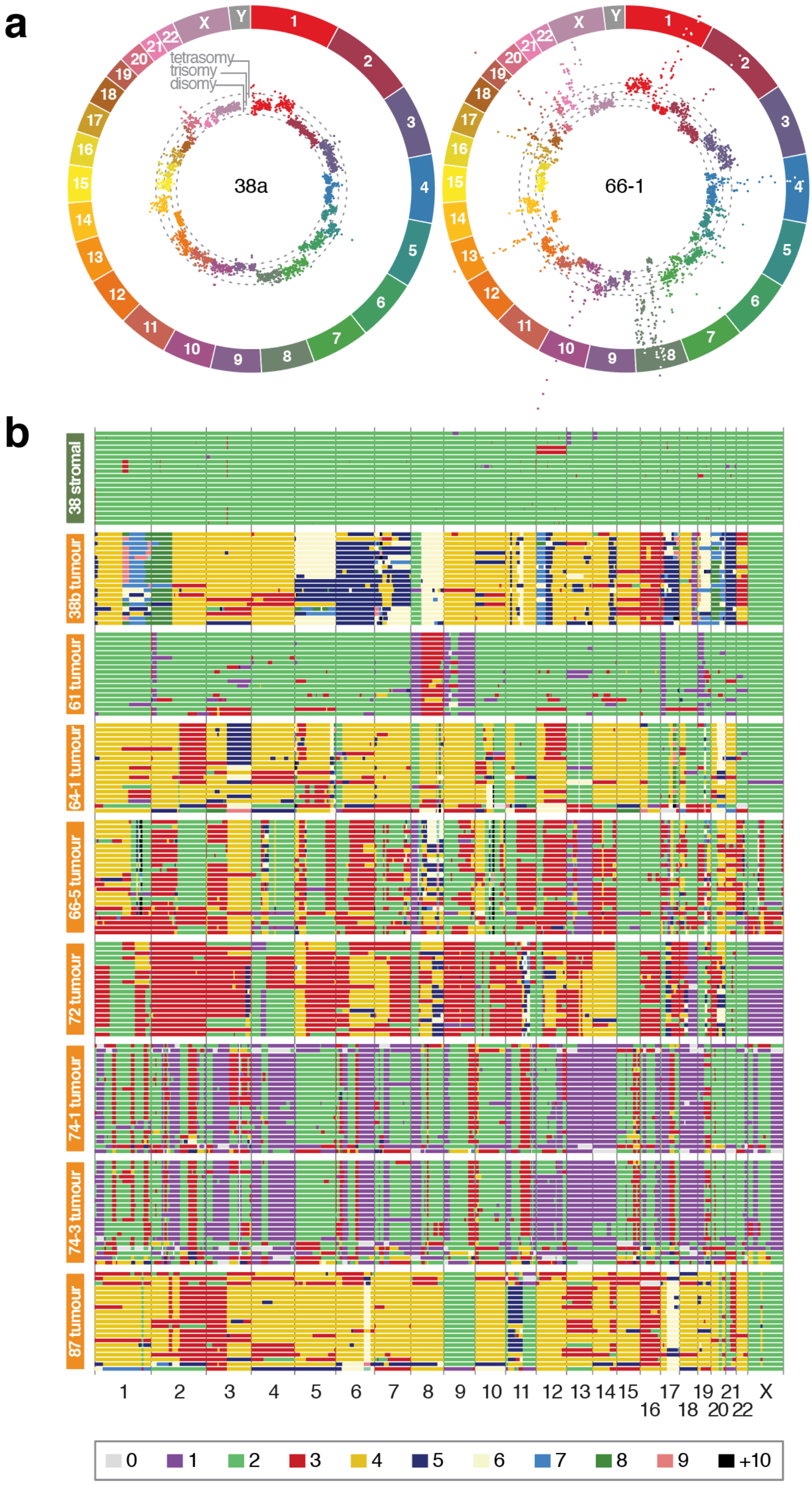

Supplementary Figure 3, related to Figure 6. scWGS karyotyping. (**a**) Circa plots for single cells showing that while the cell from OCM.38a displays chromosome arm imbalances and whole chromosome aneuploidies, the cell from OCM.66-1 also exhibits numerous focal amplifications. (**b**) Additional genome-wide chromosome copy number profiles determined by single-cell whole genome sequencing. Each row represents a single cell, with chromosomes plotted as columns and colours depicting copy number state.

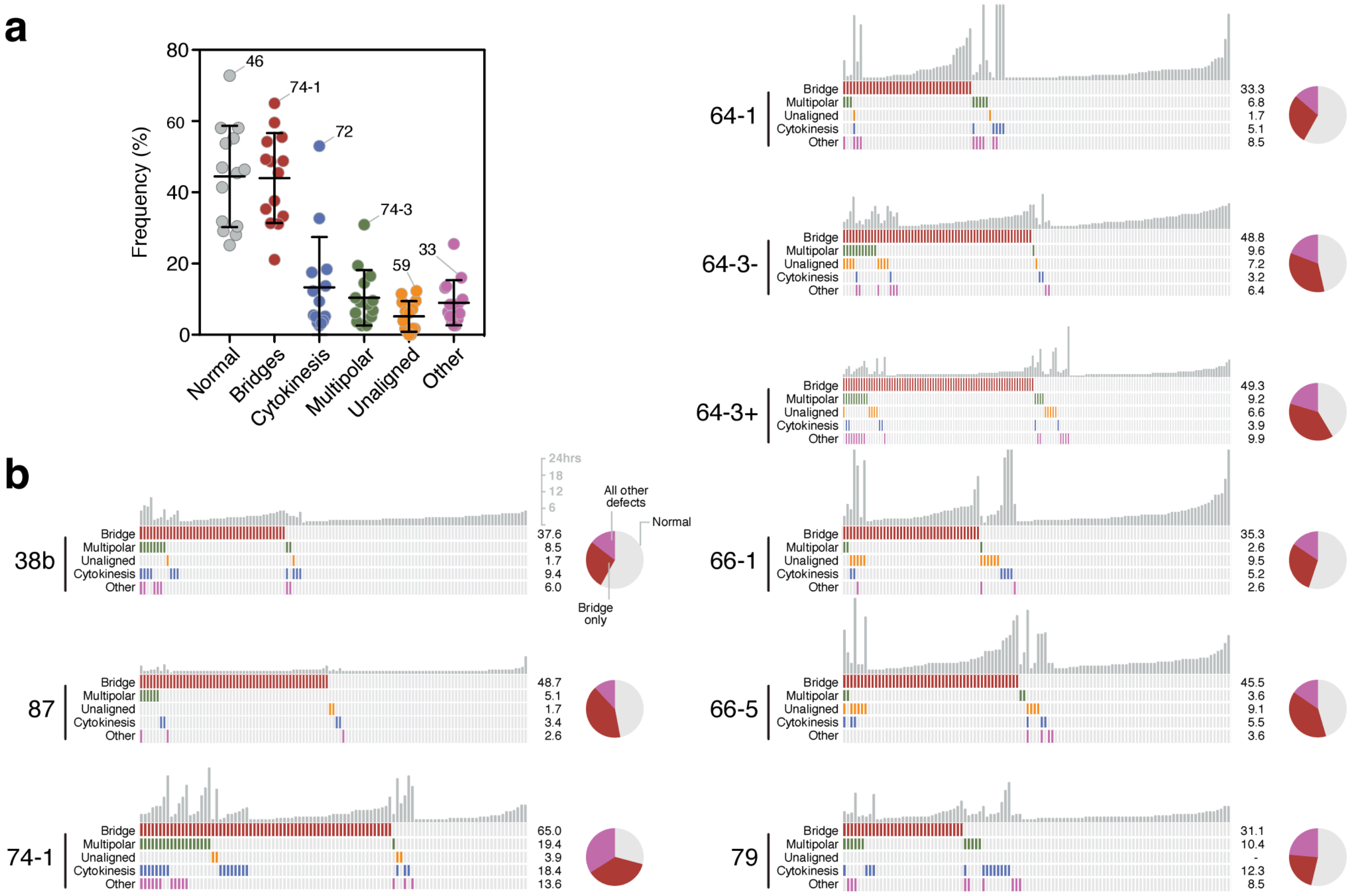

Supplementary Figure 4, related to Figure 8. Time-lapse microscopy. (**a**) Dot plot quantitating mitotic anomalies in the different models, highlighting the samples shown in Figure 8C. Bars show the mean and standard deviation (N=14 biological independent samples i.e. n=1 for each OCM). (**b**) Additional profiles, quantitating mitotic anomalies in at least 100 cells. Bars represent time each cell spent in mitosis. Source data for panels a and b are provided as a Source Data file, including number of biological independent samples for each OCM in panel b (at least 100).

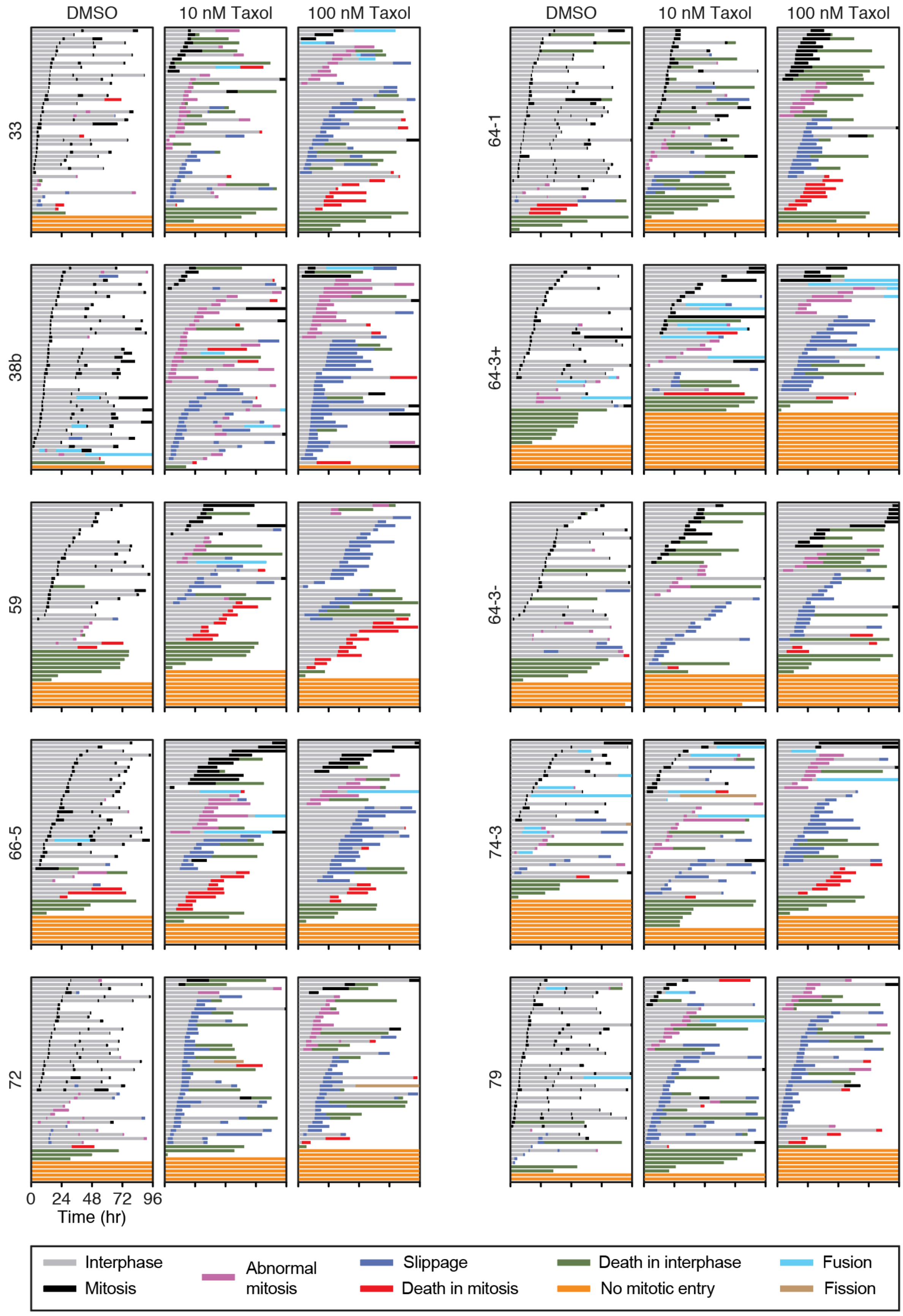

Supplementary Figure 5, related to Figure 10. Cell fate profiles. Additional cell fate profiles of untreated cultures and following exposure to paclitaxel.

Patient #	Age at dx	Anatomical site	Histology		FIGO stage	Cytoreductive Surgery	Prior lines of CTx	Platinum-response		Paclitaxel-response	
			Morphology	Grade				Radiological	CA125	Radiological	CA125
33	68	PP	Serous	3	4B	No	2	SD (C/G)	Yes	-	-
38	81	OV/PP	Serous	3	3C	No	0	PR (C/T)	Yes	PR (C/T)	Yes
46	71	OV/PP	Serous	3	3C	No	1	PD (C/T)	No	PD (C/T)	No
59	45	OV/PP	Serous	3	3C	Yes	2	PD (Cis/G)	No	-	-
61	53	OV	Mucinous	1	1C	Yes	0	CR (C)	NA#	-	-
64-1	58	OV/PP	Serous	3	3C	Yes	2	PD (C/PLD)	No	-	-
64-3-							3	-	-	Mixed (wkly T)	Yes
64-3+							3	-	-		
66-1	64	OV/PP	Serous	3	3C	No	1	PD (C/T)	No	PD (C/T)	No
66-5							1				
72	25	OV	Mucinous	2/3	1A	Yes	1	Unclear* (C/T)	Yes	Unclear* (C/T)	Yes
74-1	64	OV	Serous	3	3B	Yes	7	PR (Cis/G)	Yes	-	-
74-3							8	PR (C)	Yes	-	-
79	73	OV	Serous	3	3C	Yes	3	PD (Cis/G)	No	-	-
87	57	OV	Serous	3	3B	No	0	NA†	NA†	-	-

Supplementary Table 1. Clinical data. Table outlines demographic data and treatment responses for patients from whom the living biobank samples were taken. The platinum- and paclitaxel-responses reported are from the line of chemotherapy administered during or immediately proceeding the timepoint(s) when the ascites/solid biopsies for the living biobank were taken. Key: Asterisk, patient died before radiological assessment of anti-tumour response; Dagger, patient died before receiving chemotherapy; Hash, patient had no detectable serum CA125 throughout their illness and treatment (i.e. CA125 non-secretor); OV= ovarian; PP= primary peritoneal; CR= complete radiological response; dx= diagnosis; PD= progressive disease; PR= partial radiological response; SD= stable disease; NA= not applicable; C= carboplatin; T= paclitaxel; PLD= pegylated liposomal doxorubicin (Caelyx); Cis= cisplatin; G= gemcitabine; wkly T= weekly dose-dense paclitaxel; CTx= chemotherapy; FIGO= The International Federation of Gynecological Oncology and Obstetrics staging system.

Patient	OCM – RT-PCR			OCM – Exome			Primary tumour – targeted amplicon		
	DNA	Protein	Mutation	DNA	Protein	Mutation	DNA	Protein	Mutation
33	c.783insATT	p.262insI	Inframe insertion	c.783-1G>T	p.?	Splicesite	NA		
38	c.375_395del	p.126-132delYSPALNK	Inframe deletion	c.376-1G>C	p.?	Splicesite	c.376-1G>C	p.?	Splice site
46	c.267delC	p.S90Pfs*33	Frameshift-deletion	c.268_269delTC	p.S90Lfs*58	Frameshift-deletion	c.267delC	p.S90Pfs*33	Frameshift-deletion
59	c.398delT	p.M133Sfs*37	Frameshift-deletion	c.399_400delGT	p.M133Ifs*15	Frameshift-deletion	c.398delT	p.M133Sfs*37	Frameshift-deletion
61	c.524G>A	p.R175H	Missense	c.524G>A	p.R175H	Missense	c.524G>A	p.R175H	Missense
64	c.646G>A	p.V216M	Missense	c.646G>A	p.V216M	Missense	c.646G>A	p.V216M	Missense
66	c.488A>G	p.Y163C	Missense	c.488A>G	p.Y163C	Missense	NCC < 10%		
72	c.843C>G	p.D281E	Missense	c.843C>G	p.D281E	Missense	c.843C>G	p.D281E	Missense
74	c.1024C>T	p.R342*	Nonsense	c.1024C>T	p.R342*	Nonsense	c.1024C>T	p.R342*	Nonsense
79	c.153_162del	p.Q52Rfs*7	Frameshift-deletion	c.155_165del	p.Q52Rfs*7	Frameshift-deletion	NA		
87	WT	WT	WT	WT	WT	WT	NA		

Supplementary Table 2. *TP53* genotyping. Table summarizes *TP53* variants detected in the OCMs, by Sanger sequencing of RT-PCR products and exome analysis, and the primary tumours, by targeted amplicon sequencing of archival tumour material isolated from FFPE blocks. For each method, the DNA sequence, the predicted effect on the protein and the nature of the mutation are indicated. Note that for patients 33, 79 and 87, archival blocks were not available; for 66, the neoplastic cell count (NCC) was less than 10%.
