# Supplementary material for: A living biobank of ovarian cancer ex vivo models reveals profound mitotic heterogeneity
Source: Nat Commun. 2020 Feb 13;11:822. doi: 10.1038/s41467-020-14551-2 (PMC7018727; doi:10.1038/s41467-020-14551-2)
Supplement: Supplementary file 3 — Reporting Summary [file 41467_2020_14551_MOESM3_ESM.pdf]

nature research

Corresponding author(s): Stephen. S. Taylor

Last updated by author(s): Jan 9, 2020

Reporting Summary

Nature Research wishes to improve the reproducibility of the work that we publish. This form provides structure for consistency and transparency in reporting. For further information on Nature Research policies, seeAuthors & Referees and theEditorial Policy Checklist .

Statistics

For all statistical analyses, confirm that the following items are present in the figure legend, table legend, main text, or Methods section.

n/a	Confirmed	
☐	☒	The exact sample size (*n*) for each experimental group/condition, given as a discrete number and unit of measurement
☐	☒	A statement on whether measurements were taken from distinct samples or whether the same sample was measured repeatedly
☐	☒	The statistical test(s) used AND whether they are one- or two-sided<br>*Only common tests should be described solely by name; describe more complex techniques in the Methods section.*
☒	☐	A description of all covariates tested
☒	☐	A description of any assumptions or corrections, such as tests of normality and adjustment for multiple comparisons
☐	☒	A full description of the statistical parameters including central tendency (e.g. means) or other basic estimates (e.g. regression coefficient) AND variation (e.g. standard deviation) or associated estimates of uncertainty (e.g. confidence intervals)
☒	☐	For null hypothesis testing, the test statistic (e.g. *F*, *t*, *r*) with confidence intervals, effect sizes, degrees of freedom and *P* value noted<br>*Give P values as exact values whenever suitable.*
☒	☐	For Bayesian analysis, information on the choice of priors and Markov chain Monte Carlo settings
☒	☐	For hierarchical and complex designs, identification of the appropriate level for tests and full reporting of outcomes
☒	☐	Estimates of effect sizes (e.g. Cohen's *d*, Pearson's *r*), indicating how they were calculated

Our web collection on statistics for biologists contains articles on many of the points above.

Software and code

Policy information about availability of computer code

Data collection	Flouresence imaging: MetaMorph software (MDS Analytical Technologies); Proliferation and drug sensitivity: IncuCyte® ZOOM Analysis Software (Essen Bioscience).
Data analysis	Flouresence imaging: MetaMorph software (MDS Analytical Technologies); Genus Cytovision software (Leica); Adobe Photoshop CC 2015 (Adobe Systems Inc.). Flow cytometry: Flowjo® software (FlowJo, LLC); Proliferation and drug sensitivity; IncuCyte® ZOOM Analysis Software. Sequencing of RT-PCR products Seqman Pro (DNASTAR). Next generation sequencing and bioinformatics: bcl2fastq (Illumina); FastQC v0.11.3; FastQ Screen v0.9.2 (Babraham Institute); Trimmomatic v0.36 (Bolger et al., 2014); BWA-MEM v0.7.15 (Li and Durbin, 2010); STAR v2.4.2a (Dobin et al., 2013); Samtools v1.4 (Li et al., 2009); htseq-count v0.6.1p1 (Anders et al., 2015); DESeq2 v1.10.0 on R v3.2.3 (Love et al., 2014); Picard Tools v2.1.0; Varscan 2 (v.2.4.3) (Koboldt et al., 2013; Koboldt et al., 2012). HTSeq (v0.6.1.p1) (Anders et al., 2015); SCDE package (Kharchenko et al., 2014); PAGODA, (Fan et al., 2016); Cyclone (Scialdone et al., 2015); DAVID 6.8 (Huang da et al., 2009); REVIGO (Supek et al., 2011); Cytoscape 3.4.0 (Shannon et al., 2003); GeneMANIA (Montojo et al., 2010). FastqStrand (v. 0.0.5); Cell Ranger v3.0.1 (10x Genomics); Bowtie2 (version 2.2.4); BamUtil (version 1.0.3.); AneuFinder (version 1.4.0, Bakker et al., 2016); Circa (OMGenomics). Data presentation: Prism 7 and Prism 8 (GraphPad).

For manuscripts utilizing custom algorithms or software that are central to the research but not yet described in published literature, software must be made available to editors/reviewers. We strongly encourage code deposition in a community repository (e.g. GitHub). See the Nature Research guidelines for submitting code & software for further information.

Data

Policy information about availability of data

All manuscripts must include a data availability statement. This statement should provide the following information, where applicable:

- Accession codes, unique identifiers, or web links for publicly available datasets
- A list of figures that have associated raw data
- A description of any restrictions on data availability

Exome sequencing, RNAseq, single cell RNAseq and scWGS karyotyping data have been deposited at the EMBL-EBI with the following accession numbers E-

MTAB-7225, E-MTAB-7223, E-MTAB-724, E-MTAB-8559 and PRJEB28664 respectively. The data underlying Figs. 2b, 3a, d, 4b, c, d, e, g, 5b, e, 6b, 7a, b, d, 8b, c, 9b, c, 10a, d, S2a, S4a, b are provided as a Source Data file. All other data supporting the findings of this study are available within the article, the Supplementary information files, or the corresponding author upon request. A reporting summary for this article is available as a Supplementary Information file.

Field-specific reporting

Please select the one below that is the best fit for your research. If you are not sure, read the appropriate sections before making your selection.

☒ Life sciences ☐ Behavioural & social sciences ☐ Ecological, evolutionary & environmental sciences

For a reference copy of the document with all sections, see nature.com/documents/nr-reporting-summary-flat.pdf

Life sciences study design

All studies must disclose on these points even when the disclosure is negative.

Sample size	We describe 15 ex vivo cultures generated from 12 patients as a proof of principle cohort. Beyond that, where appropriate, sample sizes are denoted in the figure legends.
Data exclusions	During the study we realized that OCM69 was a stromal culture; rather than exclude it, we retained it to provide an additional internal control for the NGS experiments. If it became apparent that a stromal culture underwent senescence during an experiment, it was excluded and the experiment repeated with an earlier passage.
Replication	Descriptive analyses were confirmed via orthogonal approaches: e.g. TP53 mutations were identified by Sanger sequencing of RT-PCR products, exome sequencing and targeted amplicon sequencing. Doubling times and IC50 values were determined by analyzing three technical replicates and at least three biological replicates.
Randomization	There was no need to randomize samples in this study.
Blinding	For the cell biology studies the samples were not blinded. For the Exome, RNAseq, scRNAseq, scWGS, M-FISH and IHC, samples were processed and analyzed by colleagues without specifically knowing what any given sample represented.

Reporting for specific materials, systems and methods

We require information from authors about some types of materials, experimental systems and methods used in many studies. Here, indicate whether each material, system or method listed is relevant to your study. If you are not sure if a list item applies to your research, read the appropriate section before selecting a response.

Materials & experimental systems

n/a	Involved in the study	
☐	☒	Antibodies
☐	☒	Eukaryotic cell lines
☒	☐	Palaeontology
☒	☐	Animals and other organisms
☒	☐	Human research participants
☒	☐	Clinical data

Methods

n/a	Involved in the study	
☒	☐	ChIP-seq
☐	☒	Flow cytometry
☒	☐	MRI-based neuroimaging

Antibodies

Antibodies used	Brilliant Violet 421 mouse monoclonal anti-human CD44 (clone: BJ18), Biolegend, Cat no. 338810; PE/Cy7 Mouse monoclonal anti-human CD326 (EpCAM) (clone: 9C4), Biolegend, Cat no. 324222; Mouse monoclonal anti-CA125 (clone: 618F), Biolegend, Cat no. 666902; APC anti-human CD105 (clone: 43A3), Biolegend, Cat no. 323208; Zombie Yellow™ Fixable Viability Kit, Biolegend, Cat no. 423103; Goat anti-Mouse IgG (H+L) Highly Cross-Adsorbed Secondary Antibody, Alexa Fluor 488, Molecular Probes, Cat no. a11029
Validation	Brilliant Violet 421 mouse monoclonal anti-human CD44: Tyagi N, et al. 2016. Cancer Lett. 370: 260-267 and Ender F, et al. 2017. PLoS One. 12(2):e0172446. RRID: AB_2562406. PE/Cy7 Mouse monoclonal anti-human CD326 (EpCAM): Montoro DT, et al. 2018. Nature. 560:319 and Slamecka J, et al. 2017. Cell Cycle. 17:330. RRID: AB_2561506. Mouse monoclonal anti-CA125: RRID: AB_2564430 APC anti-human CD105: Liu T, et al. 2019. Front Endocrinol (Lausanne). 10:360 and Radhakrishnan S, et al. 2019. Mol Med Rep. 20:813. RRID: AB_755960 Zombie Yellow™ Fixable Viability Kit: Hole CR, et al. 2019. Nat Commun. 10:2955 and Hoppstädter J, et al. 2019. Front Immunol. 10:1634. Goat anti-Mouse IgG (H+L) Highly Cross-Adsorbed Secondary Antibody, Alexa Fluor 488: Xiong et al, 2017. Elife. 23:6 and Torres-Martínez et al, 2017. Exp Cell Res, 1:350(1). RRID: AB_138404

Eukaryotic cell lines

Policy information about cell lines

Cell line source(s)	Established cell lines: COV318, COV362 (Sigma), CAOV3 (ATCC), OVCAR3 (ATCC), Kuramochi, OVSAHO, OVMANA, OVISE, RMG1 (JCRB Cell Bank) and HCT-116 (ATCC). All OCM's were derived from patients treated at The Christie Hospital, Manchester, UK.
Authentication	All established cell lines were authenticated by the Molecular Biology Core Facility at the CRUK Manchester Institute using Promega Powerplex 21 System.
Mycoplasma contamination	All cell lines tested negative for mycoplasma.
Commonly misidentified lines (See ICLAC register)	N/A

Flow Cytometry

Plots

Confirm that:

- [x] The axis labels state the marker and fluorochrome used (e.g. CD4-FITC).
- [x] The axis scales are clearly visible. Include numbers along axes only for bottom left plot of group (a 'group' is an analysis of identical markers).
- [x] All plots are contour plots with outliers or pseudocolor plots.
- [x] A numerical value for number of cells or percentage (with statistics) is provided.

Methodology

Sample preparation	Adherent cells were incubated in Accutase to establish a single cell population. Cells were washed in PBS and transfered to FACS tubes for staining
Instrument	Novocyte 3000 (ACEA biosciences inc.)
Software	Flowjo
Cell population abundance	N/A
Gating strategy	Cells were initially gated on FSC/SSC to eliminate debris only. A mixture of cell types to ensure a population of cells expressed each of the markers was used to set up FMO's. This allowed establishment of gates for the positive and negative populations for each antibody (including a viability reagent). Once this was established, the gating strategy was first used to eliminate the debris and identify cells, followed by elimination of dead cells using the viability reagent. Using only the live cell population the percentage of positive cells for each antibody was determined using the gate established by the FMO's. These gates were also confirmed using a single stained population mixed 50:50 with unstained cells.

- [x] Tick this box to confirm that a figure exemplifying the gating strategy is provided in the Supplementary Information.
